# Low-cost computation for isolated sign language video recognition with multiple reservoir computing

**DOI:** 10.1371/journal.pone.0322717

**Published:** 2025-07-30

**Authors:** A.R. Syulistyo, Y. Tanaka, D. Pramanta, N. Fuengfusin, H. Tamukoh

**Affiliations:** 1 Graduate School of Life Science and Systems Engineering, Kyushu Institute of Technology 2-4 Hibikino, Wakamatsu, Kitakyushu, Japan; 2 Department of Information Technology, State Polytechnic of Malang, Jl. Soekarno Hatta No. 9, Lowokwaru 65141 Malang, Indonesia; 3 Research Center for Neuromorphic AI Hardware, Kyushu Institute of Technology 2-4 Hibikino, Wakamatsu, Kitakyushu, Japan; 4 Department of Information and Network Sciences, Kyushu Institute of Information Sciences, 6-3-1, Saifu, Dazaifu, Japan; Universiti Teknologi Petronas: Universiti Teknologi, MALAYSIA

## Abstract

Sign language recognition (SLR) has the potential to bridge communication gaps and empower hearing-impaired communities. To ensure the portability and accessibility of the SLR system, its implementation on a portable, server-independent device becomes imperative. This approach facilitates usage in areas without internet connectivity, addressing the need for data privacy protection. Although deep neural network models are potent, their efficacy is hindered by computational constraints on edge devices. This study delves into reservoir computing (RC), which is renowned for its edge-friendly characteristics. Through leveraging RC, our objective is to craft a cost-effective SLR system optimized for operation on edge devices with limited resources. To enhance the recognition capabilities of RC, we introduce multiple reservoirs with distinct leak rates, extracting diverse features from input videos. Prior to feeding sign language videos into the RC, we employ preprocessing via MediaPipe. This step involves extracting the coordinates of the signer’s body and hand locations, referred to as keypoints, and normalizing their spatial positions. This combined approach, which incorporates keypoint extraction via MediaPipe and normalization during preprocessing, enhances the SLR system’s robustness against complex background effects and varying signer positions. Experimental results demonstrate that the integration of MediaPipe and multiple reservoirs yields competitive outcomes compared with deep recurrent neural and echo state networks and promises significantly lower training times. Our proposed MRC achieved accuracies of 60.35%, 84.65%, and 91.51% for the top-1, top-5, and top-10, respectively, on the WLASL100 dataset, outperforming the deep learning-based approaches Pose-TGCN and Pose-GRU. Furthermore, because of the RC characteristics, the training time was shortened to 52.7 s, compared with 20 h for I3D and the competitive inference time.

## Introduction

Language serves as a vital means of communication, each with syntax and grammar [1]. Sign language, which is utilized by individuals with hearing impairments, presents a unique linguistic form. The World Health Organization (WHO) estimates that, as of 2021, 430 million people grapple with deafness [2]. Deafness extends its impact across various facets, including education, employment, social dynamics, loneliness, and stigma. Despite the universal right to equal opportunities, global disparities persist, notably in education. Communication barriers, especially for those reliant on sign language, contribute to this inequality.

Challenges arise when individuals using sign language attempt to communicate with those unfamiliar with it, hindering the smooth exchange of information [3]. Advanced technologies offer a potential solution, bridging the communication gap between hearing-impaired individuals and others. A pivotal tool in this regard is a sign language recognition (SLR) system, which processes inputs to recognize specific labels [4–6]. This study aims to develop a model requiring modest computational resources for integration into edge devices. The implementation of SLR in edge computing offers advantages such as portability, enhanced data privacy, reduced transmission costs, and usability in areas lacking internet connectivity [7].

SLR research falls into two primary categories [6]: continuous SLR, which recognizes one or more labels in continuous stream input, and isolated SLR, which identifies one sign at a time. This study specifically targets isolated SLR with low computational resource requirements. SLR categorization is based on input types, distinguishing between vision-based, sensor-based, and hybrid approaches [3,5,8]. Vision-based input involves image or video acquisition for processing the signer’s pose information. Sensor-based methods utilize wearable sensors to capture hand gestures and their positions. Hybrid approaches integrate vision-based cameras and various sensors, such as depth camera sensors. Given the user-friendly nature of vision-based approaches, particularly the minimal restraint imposed on users compared with sensor-based methods, SLR researchers predominantly emphasize vision-based systems. Calibration challenges between vision-based modalities and wearable sensors, as encountered in hybrid systems, can be particularly intricate. Considering the advantages of the vision-based approach and previous studies, this study concentrates on vision-based methodology, employing videos as input. Employing an empirical method, the SLR function uses a camera to capture signer movements, subsequently processing them further through a classification algorithm.

The domain of SLR presents a multitude of challenges, encompassing disparate video lengths, analogous gestures affiliated with distinct labels, variations in gestures within the same label [9], and the imperative aspect of real-time SLR [8]. Noteworthy endeavors have been undertaken by scholars, including Li *et al*. [9], who proposed a sizable American Sign Language video dataset, thereby contributing to a publicly accessible repository. For a parallel trajectory, Subramanian *et al*. [10] devised a streamlined approach by developing a minimized gated recurrent unit (GRU) model. This innovative model not only expedites convergence but also mitigates the computational overhead associated with the conventional GRU. Extending their contributions, Subramanian *et al*. [11] suggested the fusion of MediaPipe [12] with an optimized GRU architecture, ensuring efficient information processing. MediaPipe, an instrument created by Google, serves the purpose of constructing efficient on-device machine learning pipelines tailored for the processing of video, image, text, and audio.

The application of deep learning in SLR has been frequent owing to its inherent ability to classify both spatial and temporal features accurately. The deep learning systems applied include pose-based temporal graph convolution network (Pose-TGCN) [9], pose-gated recurrent unit (Pose-GRU) [9], inflated 3D ConvNet (I3D) [9], and MediaPipe Optimized GRU (MOPGRU) [10]. Recent studies have proposed utilizing deep neural networks (DNNs) with SLR systems. However, DNNs possess intricate architectures that heavily depend on GPUs, posing challenges in their implementation on edge devices [7] that require a significant amount of computation [13], which can lead to increased power consumption and latency. Additionally, DNNs typically require long training times, which can delay model updates [14]. To overcome these challenges, an alternative approach involving RC has been suggested [12,15–17]. RC, known for its suitability for low-cost real-time computation, holds promise for the development of machine learning hardware devices [18–21]. It is essential to underscore RC’s proficiency in classifying temporal features relevant to this area and its ability to handle multivariate features [22]. Furthermore, the hypothesis posited by Li and Tanaka suggests that the enrichment of feature representations extracted from the input can lead to improved accuracy [23]. In the context of this study, we propose the integration of multiple reservoir-based RCs (MRCs) with MediaPipe for SLR. Compared with conventional RC, MRC attains a more comprehensive feature representation, employing distinct leak rates within each reservoir to enhance learning from video input. The proposed method processes temporal input data, specifically hand and body keypoints extracted by MediaPipe from input videos. A distinctive contribution of this study lies in the integration of MediaPipe with MRC, an aspect that has not been explored in previous studies on SLR employing echo state network (ESN)-based methods.

The primary contributions of this study are as follows:

- To the best of our knowledge, this study is the first to employ RC for the task of SLR, offering a novel approach to this domain.
- We introduce an RC-based framework that demonstrates performance comparable to that of existing deep learning methods while substantially reducing the computational training time.
- The implementation is made publicly available as open-source code at https://github.com/tamukohlaboratory/MultipleReservoirComputing-MRC, promoting transparency and facilitating further research in the field.

The remainder of this paper is structured as follows: Section 2 provides an overview of related work in SLR. Section 3 elucidates the concept of RC. In Section 4, a comprehensive account of the research methodology unfolds, encompassing the utilized data and an in-depth exposition of the proposed method. Sections 5, 6, and 7 present the experimental results, discuss the results, and draw conclusions, respectively.

## Related work

The advancement of machine learning and deep learning algorithms has yielded promising results in SLR. Several studies have been conducted to solve the problem of isolated SLR. The input to the SLR can be classified into static images and videos. Through an extensive review of the literature, we identified four studies employing static images as inputs: Shah *et al*. [1], Yasumuro and Jin’no [24], Bajaj *et al*. [25], and Attia *et al*. [26]. These studies are summarized in Table 1.

**Table 1:** Summary of sign language recognition research.

Related Work	Features	Classifier	Used Labels
Shah *et al*. [1]	SURF, LBP, EOH, HOG	SVM with multiple kernels	36 Pakistan
Yasumuro and Jin’no [24]	Keypoints	SVM	41 Hiragana, 24 Alphabetic
Bajaj *et al*. [25]	Keypoint	KNN, random forest and neural network	24 American
Attia *et al*. [26]	CNN	YOLOv5x + attention methods	36 American, 66 Bangla
Li *et al*. [9]	Keypoints	TGCN	2000 American
Bilge *et al*. [6]	Spatial, temporal, text and attribute	GZSSLR	200 American
Takayama *et al*. [27]	Keypoint	SLGCN-Transformer	2000 American, 275 Japanese
Subramanian *et al*. [11]	Keypoints	MOPGRU	12 Indian, 100 American, 64 Argentinian
Luqman *et al*. [28]	Spatial features	Stack CNN and LSTM	502 arabic, 64 Argentinian
Samaan *et al*. [29]	Keypoints	RNN	10 American

Shah *et al*. [1] pioneered the development of an SLR system tailored for 36 labels within the context of Pakistan Sign Language, predominantly relying on vision modalities. Their method encompasses four distinct feature extractions, namely, speeded-up robust features (SURFs), local binary patterns (LBPs), edge-oriented histograms (EOHs), and histograms of oriented gradients (HOGs). Each feature space subsequently undergoes processing via tenfold cross-validation to ascertain the optimal kernel among linear, Gaussian, and polynomial support vector machines (SVMs) in terms of achieving the highest average accuracy. Following this, the feature space associated with a specific kernel, demonstrating the highest average accuracy, is selected as the SVM kernel to classify the output pertaining to that particular feature space.

Yasumuro and Jin’no [24] focused on the recognition of Japanese finger spelling, employing MediaPipe. Their approach involves the utilization of an SVM for the classification task as an alternative to deep learning methods [25], aiming to increase computational efficiency. Their study employed a video, processing each frame as input to recognize finger spelling, encompassing 24 labels for the alphabet and 41 labels for the hiragana datasets. Notably, the SVM-based methodology demonstrated a reduction in computation time compared with deep learning while simultaneously achieving a higher recognition rate.

Bajaj *et al*. [25] undertook a comprehensive investigation comparing three classification algorithms in the context of SLR systems: K-nearest neighbor (KNN), random forest, and neural networks. Their research explored 28 distinct preprocessing combinations with the goal of enhancing the classification algorithm. The experimental results revealed that the application of preprocessing techniques significantly improves accuracy, with the most effective combination involving rounding, shifting, and scaling. Moreover, the optimal classification algorithm identified in their study was a neural network coupled with the aforementioned preprocessing technique.

Attia *et al*. [26] innovatively developed three deep learning models based on YOLOv5x, incorporating two attention methods: squeeze-and-excitation and a convolutional block attention module for the SLR system. The dataset employed for the study comprised 36 American labels and 66 Bangla labels. The rationale behind selecting YOLOv5x, an extension of YOLOv5, as the foundational model lies in its lightweight and rapid deployment capabilities on diverse edge devices. It is crucial to note, however, that this model necessitates bounding box labeling, rendering it trainable but requiring a considerable time investment for annotation.

As shown in Table 1, three of the four studies that utilized static images employed classical machine learning, whereas one study used deep learning. Notably, considerable emphasis has been placed by researchers on optimizing the computation time of SLR systems. Importantly, the practical application of SLR involves the analysis of videos to identify labels on the basis of motion sequences. Consequently, this study intentionally abstained from the use of static images, aligning with the dynamic nature inherent in SLR applications. The challenge encountered in the isolated SLR of video inputs revolves around the scarcity of publicly available datasets. This predicament was effectively addressed by Li [8] through the introduction of the Word-Level American Sign Language (WLASL) video dataset. The notable features of this dataset include a frame rate of 25 frames per second (fps) and a video resolution of 256×256. Ambiguity emerges as a notable challenge within WLASL. This ambiguity manifests in instances where identical sign language labels exhibit different signs. Furthermore, diverse sign languages may possess distinct labels, such as “wish” and “hungry", while featuring similar signs or movements [8]. Li proposed a method designed for recognizing isolated sign language, denoted as pose-based temporal graph convolution networks (Pose-TGCNs). This method relies on OpenPose [21] for extracting keypoints, encompassing 13 upper bodies and 21 joint points for both the left and right hands. Remarkably, the Pose-TGCN demonstrates commendable performance, particularly when confronted with a limited vocabulary size of 100 labels.

Bilge *et al*. [6] presented an SLR system designed to identify novel classes through knowledge transfer from the training dataset, specifically addressing zero-shot learning sign language recognition (ZSSLR) and generalized ZSSLR (GZSSLR). The authors employed a zero-shot learning (ZSL) framework to extend the recognition model’s applicability to both seen and unseen classes, incorporating visual and auxiliary class representations. ZSSLR and GZSSLR share similarities, differing only in the test data utilized: ZSSLR for novel, unseen test data and GZSSLR for both novel, seen, and unseen test data. Visual representations were extracted from the spatiotemporal deep model encompassing body and hand regions. An auxiliary class representation was derived from textual dictionary definitions and attribute combinations. The authors introduced three benchmark datasets in this study: ASL-Text, comprising 250 labels; and MS-ZSSLR-W and MS-ZSSLR-W, each containing 200 labels. Despite promising results, the accuracy, although relatively low compared with that of other ZSL methods, remained below 40%.

Takayama *et al*. [27] extended batch normalization in deep learning to insert masked batch normalization (MBN) in an existing SLR system. The MBN normalized the input features in the GCN model while masking the dummy signals. The experimental outcomes revealed a noteworthy enhancement in the accuracy of the GCN, establishing MBN as an effective classification algorithm. In the context of this study, the most proficient algorithm identified was a Sign Language Graph Convolution Network with a Transformer (SLGCN-Transformer). This algorithm exhibited superior performance within the experimental framework.

Subramanian *et al*. [11] directed their research toward Indian SLR involving 12 distinct classes. The authors introduced an optimized fusion of MediaPipe and a GRU, denoted as the MOPGRU (MediaPipe Optimized Gated Recurrent Unit), designed to process video datasets effectively. Within the MOPGRU, modifications were applied to the updated gates of the standard GRU, ensuring that the outputs of the reset gates re-evaluated the information, eliminating unwanted data and prioritizing meaningful information. Furthermore, the method proposed by the researchers underwent a comparative analysis with a state-of-the-art algorithm employing WLASL100 (Word Large American Sign Language with 100 labels).

Luqman *et al*. [28] devised an SLR model that synergistically employs a convolutional neural network (CNN) and long short-term memory (LSTM). This integration was evaluated via datasets comprising 502 Arabic and 64 Argentinian samples. The optimal configuration was identified through the utilization of stacked MobileNet for feature extraction, followed by subsequent processing with stacked LSTM. This combination emerged as the most effective in achieving the desired outcomes in their experimental framework.

Samaan *et al*. [29] introduced the dynamic sign language (DSL) 10 dataset, a dataset comprising 10 labels of ASL. Their approach involves the application of RNN-based models, such as GRU, LSTM, and BiLSTM.

All six studies focused on video inputs, as outlined in Table 1, and employed deep learning methodologies. According to the experimentation conducted by Samaan *et al*. [29], the use of facial keypoints is not advised because of the sixfold increase in processed features, leading to heightened computational demands. This results in extended processing times compared with scenarios where facial keypoints are not employed, while the achieved accuracy remains comparable. Similarly, other researchers [11,24,26], and [29] also consider the computational efficiency of SLR, acknowledging its significance in ensuring streamlined processing. The collective findings from SLR research underscore real-time implementation on edge devices as an ongoing challenge within SLR systems. This exploration drives our research efforts, with a focus on developing a cost-effective SLR solution applicable to edge devices adept at classifying dynamic inputs. Furthermore, our proposed method combines computational efficiency and competitive performance, unlike deep learning methods, which often demand computational power and training time.

## Reservoir computing

### ESN

**Fig 1:**
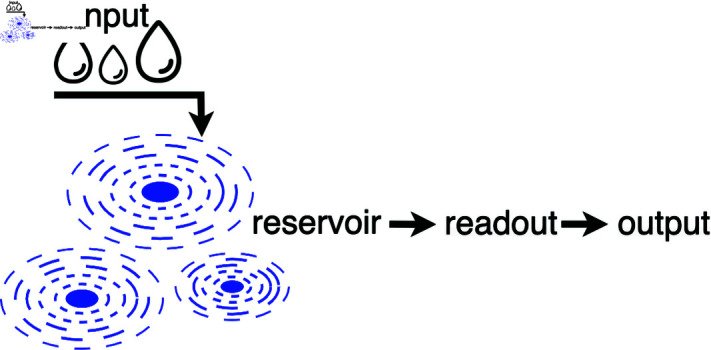
Reservoir concept depicted with ripples.

RC is inspired by a natural phenomenon: when a droplet of water falls onto a still water surface, it generates ripples that spread outward. The pattern and intensity of these ripples are determined by the size and force of the droplet, as illustrated in Fig 1. Therefore, observing the water surface can analyze what or how droplets have fallen.

**Fig 2:**
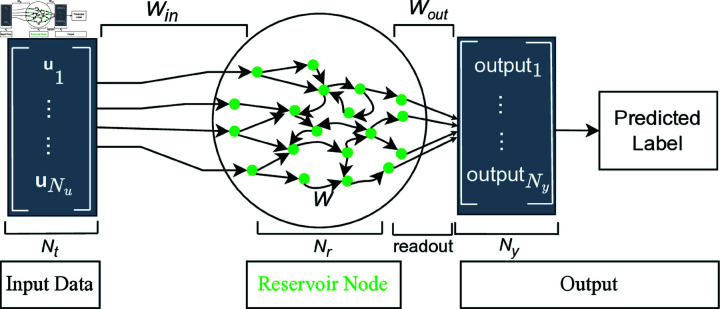
Basic architecture of ESN.

RC consists of input, reservoir, and output, as shown in Fig 2. The water surface can be regarded as an analogy for the reservoir, with the droplet representing the input signal. As the droplet interacts with the water, it disturbs the surface and generates a complex ripple pattern, analogous to how input time series data are transformed by the dynamic reservoir in RC. The reservoir captures temporal dependencies and maps the input into a high-dimensional space called a reservoir state. In the final stage of the model’s development, the readout employs the transformed states, or ripple patterns, to construct the model and perform classification.

RC presents a recurrent model capable of training without relying on a gradient descent-based approach. This design seeks to overcome the challenges associated with RNNs, which are known for being challenging to train via gradient descent methods and computationally intensive [30]. In the RC architecture, input data undergo processing within a fixed random internal layer known as the reservoir, and the output is generated through a linear combination, often implemented as linear regression [12]. Compared with the deep learning approach, this methodology enables RCs to achieve faster computation times with fewer parameters [31].

RC encompasses two primary types: ESNs [17] and liquid state machines (LSMs) [32]. The primary distinction lies in the implementation of the neurons. ESN utilizes discrete dynamics and rate-coded neurons that integrate inputs and recurrent connections, whereas LSM employs continuous dynamics and spiking neurons. This study focuses predominantly on the ESN approach because of its simplicity and robust theoretical foundation [33]. The fundamental architecture of ESN is depicted in Fig 2 and comprises four steps:

1. Generate an input weight *W*_*in*_ via Eq (1), reservoir weight *W* via Eq (4), and leak rate α, scaling in the range [0,1], which controls the effect of reservoir states at the previous timestep to the next reservoir state. Let *N*_*u*_ and *N*_*r*_ denote the dimensions of the input and reservoir vectors, respectively. $W_{in}\in {𝐑}^{N_{r}\times N_{u}}$ represents weight matrices of the input data, scaling in the range [−σ,σ]. $W\in {𝐑}^{N_{r}\times N_{r}}$ denotes weight matrices of the internal neurons, which are generated via Eqs (2), (3) and (4).
  $$W_{in}=(2\;randomBinomial(N_{r},N_{u})-1)\;\sigma ,$$ (1)
  $$W_{0}=random(N_{r},N_{r},\theta ),$$ (2)
  $$\rho_{0}=max(|eigen(W_{0})|),$$ (3)
  $$W=W_{0}(\rho /\rho_{0})$$ (4)
  Here, $randomBinomial(N_{r},N_{u})$ represents a random function, which extracts a sample from the binomial distribution to generate a matrix $\in {𝐑}^{N_{r}\times N_{u}}$.  σ represents the input scaling hyperparameter, which controls the influence of the input in the dynamic reservoir. $random(N_{r},N_{r},\theta )$ represents a sparse random function that generates a matrix in a certain dimension on the basis of the reservoir dimension $N_{r}\;\times \;N_{r}$ and the parameter θ as a connectivity value, which represents the percentage of nonzero values in the reservoir that has a value in the range of [0,1]. ρ represents the spectral radius hyperparameter, which defines the maximum absolute eigenvalue of the reservoir weight matrix, and *eigen*(*W*_0_) is a function for calculating eigenvalues on the basis of a random matrix that is generated via Eq (2).
2. Process the input **U** and calculate the corresponding reservoir activation states *x*_(*t*)_. We define the input and reservoir activation states in Eqs (5) and (6), respectively, as follows:
  $$U=[u_{\left(1\right)},u_{\left(2\right)},u_{\left(3\right)},...,u_{\left(N_{t}\right)}]\in {𝐑}^{N_{t}\times N_{u}},$$ (5)
  where *N*_*t*_ represents the time length of the input data.
  $$x_{\left(t+\Delta t\right)}=(1-\alpha )x_{\left(t\right)}+\alpha func(Wx_{\left(t\right)}+W_{in}u_{\left(t+\Delta t\right)}),$$ (6)
  where $u_{\left(t+\Delta t\right)}$ represents the input data, *x*_(*t*)_ represents the reservoir state, *t* represents the discrete time (1,2..., T), *func* represents an activation function, which typically uses a hyperbolic tangent.
3. Compute the linear readout weights *W*_*out*_ from the reservoir using linear regression. In this study, we used ridge regression, which minimizes the error between *Y*_(*t*)_, the predicted label at time *t*, and the actual label *Y*_*target*_, as defined in Eq (7), while preventing overfitting via Eq (8).
  $$Y_{target}=[y_{target(1)},y_{target(2)},...,y_{target(N_{t})}]\in {𝐑}^{N_{t}\times N_{y}},$$ (7)
  where *N*_*y*_ represents the number of dimensions of a target vector.
  $$W_{out}=(X^{⊤}X+\beta_{1}𝐼{)}^{-1}X^{⊤}Y_{target},$$ (8)
  where $\beta_{1}$ represents the regularization coefficient, $𝐼\in {𝐑}^{N_{r}\times N_{r}}$ represents the identity matrix, and $X\in {𝐑}^{N_{t}\times N_{r}}$ represents the reservoir state vector $X=\left[x_{\left(1\right)},x_{\left(2\right)},...,x_{\left(N_{t}\right)}\right]$.
4. The trained network is used on new input data **U** for computing the predicted label $Y\in {𝐑}^{N_{t}\times N_{y}}$ by utilizing the trained output weights $W_{out}\in {𝐑}^{N_{r}\times N_{y}}$, which can be formulated by using Eq (9).

$$Y=XW_{out}$$ (9)

### Grouped ESN

**Fig 3:**
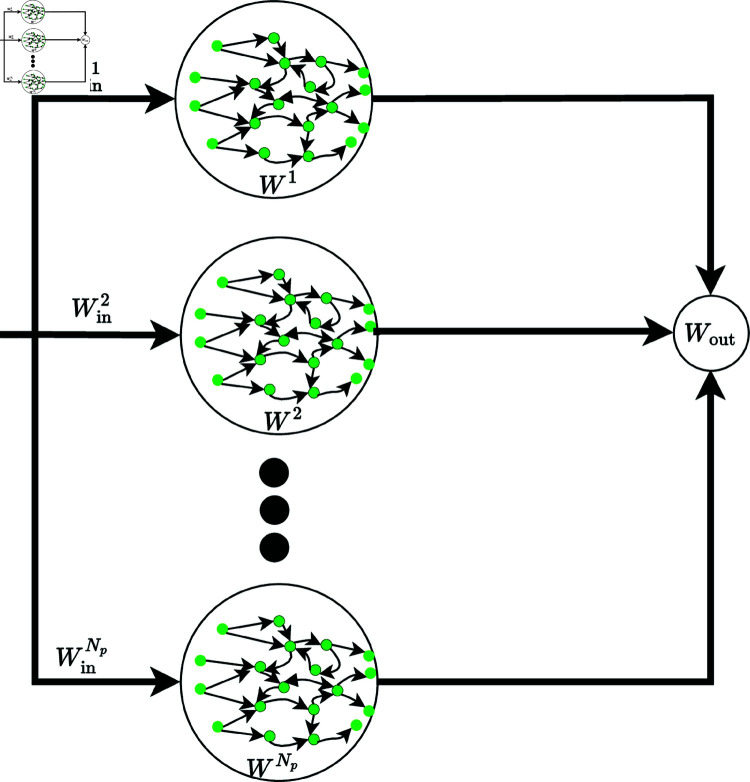
Illustration of grouped ESNs.

GroupedESN [34,35], and [36] comprise more than one parallel reservoir, denoted as *N*_*p*_, and a single linear readout serves as the decoder, as illustrated in Fig 3. This approach is introduced to extract diverse features from time series inputs, enhancing prediction performance by expanding the reservoir state space to augment its representational capabilities. The corresponding reservoir state can be computed via Eq (10) [34]. In the grouped ESN, a constant leak rate is employed to calculate the reservoir state, with independent *W*_*in*_ and *W* values for each reservoir.

$$x_{\left(t+\Delta t\right)}^{p}=(1-\alpha )x_{\left(t\right)}^{p}+\alpha func(W^{p}x_{\left(t\right)}^{p}+W_{in}^{p}u_{\left(t+\Delta t\right)}),$$ (10)

where *p* represents the index of a parallel reservoir. ${\text{W}}^{\text{p}}$ and $W_{in}^{p}$ have the same generation and distribution as in the ESN, as obtained via Eqs (1) and (4).

### Reservoir state representation

In this study, we drew inspiration from the ESN implementation proposed by Bianchi *et al*. [23]. In their implementation, they used drop parameters δ, which are used to set the length of the timestep that will be processed in the training by dropping a certain reservoir state timestep, as formulated in Eq (11). The δ parameter is useful in omitting timesteps that do not significantly contribute to the recognition process. The result of the dropping timestep is denoted as $X_{drop}\in {𝐑}^{N_{d}\times N_{r}}$, where *N*_*d*_ is the number of timesteps after the drop process on the drop value δ.

$$X_{drop}=X[0:N_{t}-\delta ,0:N_{r}],$$ (11)

where in the formula, the notation *[*0:*N*_*r*_*]* is defined as a slice of a range starting from zero and ending at *N*_*r*_−1.

We also adopt the reservoir state representation module shown in Eq (13), which is represented by *s*. This module utilizes all reservoir dynamics, in contrast to the standard ESN approach, which employs the final reservoir state because the utilization of the final state may introduce bias in the output modeling space. The other objective of this module is to increase the generalization capacity of reservoirs that rely on heterogeneous dynamics arising from inputs. Bianchi *et al*. [23] developed a new model space in which each multivariate time series is represented by linear model parameters. The linear model is trained to predict the subsequent reservoir state denoted as *x*_(*t* + 1)_ by employing the mathematical Eq (12). *s* is a vector of length *N*_*rep*_, where *N*_*rep*_ is equal to the number of rows $(N_{r}\;+\;1)N_{r}$. The notation $V=Concat{\left(V_{1}^{⊤},v_{2}^{⊤}\right)}^{⊤}$ represents a matrix resulting from the concatenation result of a weight matrix $V_{1}\in {𝐑}^{N_{r}\times N_{r}}$ and vector $v_{2}\in {𝐑}^{N_{r}}$. *V*, represented by Eq (17), denotes the outcome of the ridge regression of $X_{2}\in {𝐑}^{\left(N_{d}-1)\times (N_{r}+1\right)}$ on Eq (15), where $X_{next}\in {𝐑}^{(N_{d}-1)\times N_{r}}$ on Eq (16) serves as the target. *X*_2_ is formed by concatenating $X_{prev}$ in Eq (14) with one that is biased for the input. $v_{2}$ serves as a bias to adjust the regression line to fit the data. *V* in Eq (17) and *W*_*out*_ in Eq (8) have different purposes, despite both equations utilizing ridge regression in their process. Eq (17) is employed to use all of the reservoirs by training a linear model to predict the subsequent state of the reservoir in each timestep. By contrast, Eq (8) is used to train the model to predict the outputs of given tasks.

$$x_{\left(t+1\right)}=x_{\left(t\right)}V_{1}+v_{2},$$ (12)

$$s=vec(V)=Concat(vec(V_{1}),v_{2}),$$ (13)

where

$$X_{prev}=X_{drop}\left[0:N_{d}-1,0:N_{r}\right],$$ (14)

$$X_{2}=Concat(X_{prev},1),$$ (15)

$$X_{next}=X_{drop}\left[1:N_{d},0:N_{r}\right],$$ (16)

$$V=(X_{2}^{⊤}X_{2}+\beta_{2}{𝐼}_{2}{)}^{-1}X_{2}^{⊤}X_{next},$$ (17)

where *Concat*(.) is the concatenation function used to join a sequence of arrays with the same shape along an existing axis. The vectorization function, designated as *vec*(.), is employed to transform a matrix into a column vector, whereby the columns of the matrix are stacked in a vertical configuration. $\beta_{2}$ is the regularization parameter for ridge regression, and ${𝐼}_{2}$ is the identity matrix.

The utilization of *s* in the place of the standard reservoir state requires the modification of the readout designated as $\hat{W}\in {𝐑}^{N_{rep}\times N_{y}}$ and the predicted label designated as $\hat{Y}\in {𝐑}^{N\times N_{y}}$, as demonstrated in Eqs (18) and (19).

$$\hat{W}=(S^{⊤}S+\beta_{3}{𝐼}_{3}{)}^{-1}S^{⊤}Y_{rep},$$ (18)

$$\hat{Y}=S\hat{W},$$ (19)

where $S\in {𝐑}^{N\times N_{rep}}$ represents $S=\left[s_{\left(1\right)},s_{\left(2\right)},...,s_{\left(N\right)}\right]$, where *N* is the number of data. $\beta_{3}$ represents the regularization coefficient, ${𝐼}_{3}\in {𝐑}^{N_{rep}\times N_{rep}}$ represents the identity matrix, and $Y_{rep}\in {𝐑}^{N\times N_{y}}$ is the target matrix.

## Research method

### Data acquisition

This study employs sign language videos as input data. Subsequently, MediaPipe is employed to extract keypoints from the video dataset for each frame. The extracted keypoints encompass the body, left hand, and right hand, collectively amounting to 150 features. More precisely, 66 features pertain to the body, and 42 features each are dedicated to the left and right hands. The dataset utilized in this study is WLASL100, encompassing 100 distinct labels.

### Processing each video frame

**Fig 4:**
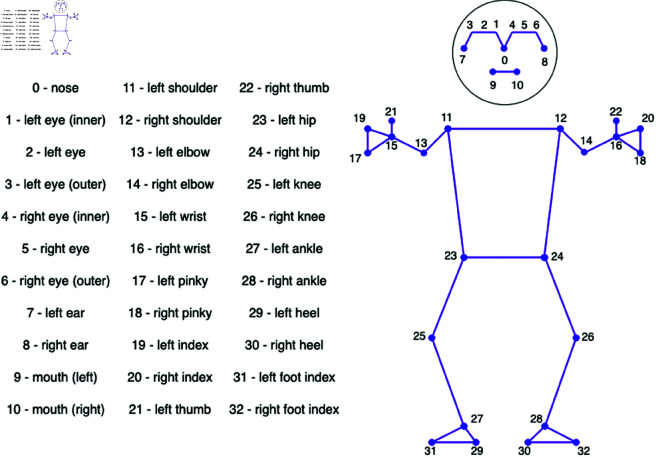
Illustration of pose landmarks.

The processing of each frame involves a two-step procedure: preprocessing and extracting keypoints through the utilization of MediaPipe. Data preprocessing plays a pivotal role in this research, as variations in the video dataset conditions can impact the accuracy of the classification algorithm. To address this, a preprocessing technique, namely, normalization and zero padding, is employed. Normalization plays a crucial role in accommodating the diverse positions of signers, using the nose position as a reference for each signer. The process involves several steps. Initially, the nose is detected as a reference point located at index 0 in the pose landmark, as illustrated in Fig 4. If the pose is not detected in certain frames, those frames are subsequently removed. The nose is chosen as a reference because its point is relatively stable and not affected by hand movement, and this point is appropriate when the head is stable. The next step involves mapping the keypoints into image coordinates, followed by subtracting all keypoints by the nose coordinate, termed the distance keypoint $dKeypoint\;\in \;{𝐑}^{N_{f}}$, as expressed in Eq 20. The mean of the *dKeypoint* is subsequently computed, resulting in $meanKeypoint\;\in \;{𝐑}^{N_{f}}$, as demonstrated in Eq 21. This value is then subtracted from *dKeypoint* via Eq 23. In the final step, as per Eq 22, the normalization result $u_{normalized}\in {𝐑}^{N_{f}}$ is obtained by dividing *meanKeypoint* by its standard deviation, computed through Eq 24.

dKeypoint=allKeypoint−nosePosition, (20)

$$meanKeypoint=dKeypoint-\overset{―}{dKeypoint},$$ (21)

$$u_{normalized}=meanKeypoint/std(meanKeypoint),$$ (22)

where

$$\overset{―}{input}=\sum (input)/N,$$ (23)

$$std(input)=\sqrt{\frac{1}{N-1}\sum_{i=1}^{N}(input_{i}-\overset{―}{input}{)}^{2}},$$ (24)

*N* represents a number of inputs, and *u*_*normalized*_ represents one timestep that will be combined for all timesteps from one video to become $U_{normalized}\;\in \;{𝐑}^{N_{t}\times N_{f}}$.

This study also explored an alternative normalization approach using the shoulder position as the reference point. The shoulder is chosen as a reference point because sign language primarily involves the upper body and hand so that it can ensure hand position alignment for SLR. The normalization process is performed by computing the center point of the shoulders via Eq 25. The length of the shoulder is then calculated via Eq 26. In the final step, *allKeypoint*, which combines hand and pose landmarks, is normalized via Eq 27.

$$middle=\frac{leftShoulder+rightShoulder}{2}$$ (25)

leftShoulder and rightShoulder represent the x and y coordinates of the left and right shoulder positions, respectively.

lScale=||leftShoulder−rightShoulder|| (26)

||.|| denotes the norm or absolute function.

$$sNormalize=\frac{allKeypoint-middle}{lScale}$$ (27)

By introducing another preprocessing technique, zero padding, denoted as $U_{padding}\in {𝐑}^{N_{t}+padding\times N_{f}}$, is performed subsequent to normalization. This step is implemented to standardize the length of the video timesteps across datasets, ensuring uniformity in temporal dimensions. Both normalization and zero padding are integral components of both the training and testing processes. In addition to these techniques, an extra preprocessing step, exclusively employed during training, is incorporated, termed augmentation. Augmentation is crucial in addressing specific challenges encountered in sign language videos, where signers predominantly employ either the left or right hand. To mitigate this bias, horizontal flipping is applied in this study. By doing so, the classification algorithm is adept at learning and adapting to scenarios where the signer predominantly uses either the left or right hand.

### Proposed methods

**Fig 5:**
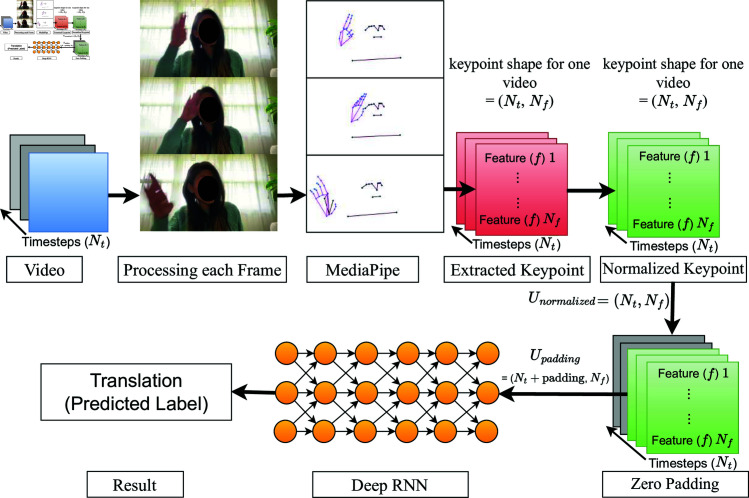
Sign language recognition pipeline.

**Fig 6:**
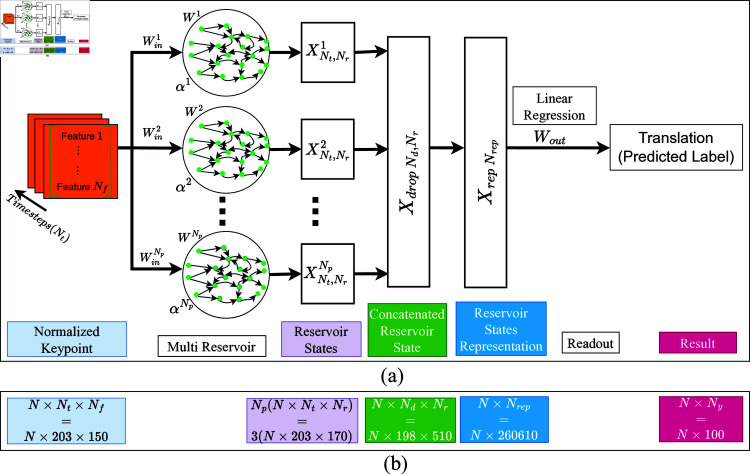
a) Reservoir computing based on multiple reservoirs. b) Illustration of matrix size on MRC510.

This study introduces a novel approach, termed MRC, that integrates MediaPipe into the SLR pipeline, as illustrated in Fig 5. Preceding the RC processing step, feature normalization and zero padding are executed, involving the calculations outlined in Eqs (20), (21), and (22). The preprocessed features are then fed into the MRC, as depicted in Fig 6(a), employing distinct leak rates α for each reservoir. The parallel reservoirs, denoted by the index representation *p*, calculate the reservoir state via Eq 28. The influence of the previous state on the current state varies on the basis of the leak rate; a lower rate implies a more significant influence, whereas a higher rate results in less impact. This diversification in reservoir characteristics within the MRC facilitates the extraction of distinct signing speeds, contributing to a richer data representation than a conventional RC. The reservoir states from all the reservoirs in the MRC are aggregated, and the resulting representation is further processed through Eq 13. Subsequently, linear regression is applied for training or inference via Eq 18.

Algorithm 1 presents the pseudocode for training the MRC, whereas Algorithm 2 outlines the pseudocode for inference. Throughout the training and inference processes, various functions come into play. Specifically, *generateInternalWeight*(.) is utilized to generate *W*, as illustrated in Eq 4. Additionally, the function *generateInputWeight*(.) is employed to create *W*_*in*_ following Eq 1. The function *reservoirState*(.) is invoked to calculate the reservoir state, as indicated in Eq 28. Furthermore, the function *s*(.) is employed for computing the reservoir representation, as depicted in Eq 13. The function *TrainRegression* is utilized to train the reservoir weight, following Eq 18.

$$x_{\left(t+\Delta t\right)}^{p}=(1-\alpha^{p})x_{\left(t\right)}^{p}+\alpha^{p}func(W^{p}x_{\left(t\right)}^{p}+W_{in}^{p}u_{\left(t+\Delta t\right)})$$ (28)

The weights generated in the training process outlined in Algorithm 1 are subsequently employed to predict the labels *Y* of the test data, as detailed in Algorithm 2. This process involves utilizing the *loadTrainingInternalWeight*() function for *W*_*in*_, *loadTrainingInputWeight*() for *W*, and the readout weight $\hat{W}$.

**Algorithm 1.** Training process of MRC

**Input** Input data matrix **U**, input data on the *t* timestep *u*_(*t*)_, target data matrix *Y*_*rep*_, internal unit number of reservoir *N*_*r*_, number of parallel reservoirs *N*_*p*_, leaking rate for each reservoir $\alpha_{p}$, spectral radius ρ, connectivity θ, input scaling σ, weight matrices of the internal neurons *W*, weight matrices of input data *W*_*in*_, time length of input data *N*_*t*_, and the number of reservoir states to be dropped δ

**Output** decoding module $\hat{W}$

1: **for** *p* = 1 to *N*_*p*_ **do**

2:   $W[p]=generateInternalWeight(N_{r},\rho ,\theta )$

3:   $W_{in}[p]=generateInputWeight(U,N_{r},\sigma )$

4: **end for**

5: **for** *p* = 1 to *N*_*p*_ **do**

6:   **for** t = 0 to *N*_*t*_−1 **do**

7:    $x_{\left(t+\Delta t\right)}^{p}=reservoirState(\alpha_{p},W[p],x_{\left(t\right)}^{p},W_{in}[p],u_{\left(t+\Delta t\right)})$

8:   **end for**

9:   $X=Concat(x_{\left(t\right)}^{p},x_{\left(t+1\right)}^{p},...,x_{\left(N_{t}-1\right)}^{p})$

10:   $X_{drop}[p]=X[:N_{t}-\delta ,:N_{r}]$

11:   **if** *p* = 1 **then**

12:    *allX* = *X*_*drop*_*[p]*

13:   **else**

14:    *allX* = *ColumnStack*(*allX*,*X*_*drop*_*[p]*)

15:   **end if**

16: **end for**

17: S=Concat(s(allX[0]),...,s(allX[N]))

18: $\hat{W}$ = *TrainRegression*(*S*,*Y*_*rep*_)

19: **return** $\hat{W}$

**Algorithm 2.** Inference process of MRC

**Input** input data matrix **U**, input data on the *t* timestep *u*_(*t*)_, internal unit number of reservoir *N*_*r*_, number of parallel reservoirs *N*_*p*_, leaking rate for each reservoir $\alpha_{p}$, weight matrices of the internal neurons *W*, weight matrices of input data *W*_*in*_, time length of input data *N*_*t*_, trained output weights $\hat{W}$, and the number of reservoirs state to be dropped δ

**Output** Prediction label $\hat{Y}$

1: W=loadTrainingInternalWeight()

2: *W*_*in*_ = *loadTrainingInputWeight*()

3: $\hat{W}=loadTrainingOuputtWeight()$

4: **for** *p* = 1 to *N*_*p*_ **do**

5:   **for** t = 0 to *N*_*t*_−1 **do**

6:    $x_{\left(t+\Delta t\right)}^{p}=reservoirState(\alpha_{p},W[p],x_{\left(t\right)}^{p},W_{in}[p],u_{\left(t+\Delta t\right)})$

7:   **end for**

8:   $X=Concat(x_{\left(t\right)}^{p},x_{\left(t+1\right)}^{p},...,x_{\left(N_{t}-1\right)}^{p})$

9:   $X_{drop}[p]=X[:N_{t}-\delta ,:N_{r}]$

10:   **if** *p* = 1 **then**

11:    *allX* = *X*_*drop*_*[p]*

12:   **else**

13:    *allX* = *ColumnStack*(*allX*,*X*_*drop*_*[p]*)

14:   **end if**

15: **end for**

16: S=Concat(s(allX[0]),...,s(allX[N]))

17: $\hat{Y}$ = $S\hat{W}$

18: **return** $\hat{Y}$

## Experiments

### Experimental setting

The SLR experiment was conducted using Python version 3.10 on a personal computer featuring an Intel Core i7 central processing unit (CPU), 32 GB of random access memory (RAM) and a 12 GB NVIDIA GeForce RTX 4070 Ti graphics processing unit (GPU). The WLASL100 dataset was partitioned into three segments, training, validation, and testing, comprising 1780 videos, 258 videos, and 258 videos, respectively.

The proposed MRC encompasses two distinct architectural configurations, each comprising 300 and 510 reservoir nodes. The aforementioned architectures are composed of either two or three parallel reservoirs. The leakage rates applied in each reservoir vary to enhance temporal feature extraction. The values are set at 0.9 for the first reservoir, 0.8 for the second reservoir, and 0.6 for the third reservoir in the three-reservoir configuration. Furthermore, a parameter of 0.3 is assigned for the spectral radius ρ, which determines the largest value of the absolute eigenvalue of the reservoir. Other key parameters include five for the number of reservoir states to be dropped δ, 0.2 for the connectivity value θ, and $\beta_{2}$ (15 for *V* in Eq 17)) and regularization coefficients of $\beta_{3}$ (3 for $\hat{W}$ in Eq 18). Both coefficients utilize the ridge regression algorithm. These values are obtained from a hyperparameter optimization framework, Optuna [37]. The search space for each hyperparameter is shown in Table 2.

**Table 2:** Hyperparameter value range search space.

Hyperparameter	Symbol	Value
Leak rate	α	0.1 to 1
Spectral radius	ρ	0.1 to 1
Connectivity	θ	0.1 to 1
Reservoir state representation regularization coefficient (w_ridge)	$\beta_{2}$	1 to 30
Readout regularization coefficient (w_ridge_embedding)	$\beta_{3}$	1 to 30
Drop reservoir	δ	1 to 10
Input scaling	σ	0.1 to 1

**Fig 7:**
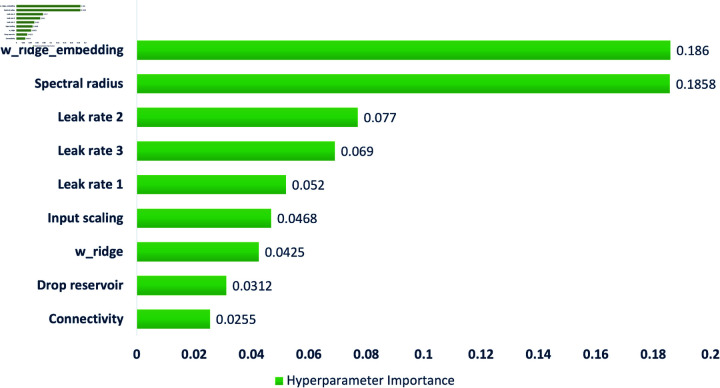
Importance of hyperparameter.

The hyperparameter importance analysis in Fig 7 shows the average result of the Optuna hyperparameter importance values during fine-tuning from 10 optimization runs and 30 trials for each run. The optimization runs reveal that w_ridge_embedding ($\beta_{2}$) has the most significant impact on model performance, indicating that controlling $\beta_{2}$ in training is crucial for improving generalization. Similarly, the spectral radius ρ contributes almost equally, suggesting that both parameters play a key role in model stability and feature transformation. The leak parameters α (leak rates 1 ($\alpha_{1}$), 2 ($\alpha_{2}$), and 3 ($\alpha_{3}$) play a significant yet secondary role, indicating that fine-tuning them could optimize memory and state propagation in reservoir computing. Moreover, input scaling (σ) has a noticeable but lower influence, meaning that it affects model sensitivity but is not as critical as the other parameters. On the other hand, w_ridge ($\beta_{3}$), the drop reservoir (δ), and connectivity (θ) have minimal impacts, suggesting that their tuning is less critical and that default values may be sufficient.

In Fig 6(b), the worst-case experimental scenario for matrix operations in this research is illustrated. The MRC170*3 (MRC510) configuration, comprising three reservoirs with 170 nodes each, results in a total of 510 nodes in the reservoir. Here, *N* represents the number of matrix samples, *N*_*t*_ denotes the number of timesteps, *N*_*f*_ represents the number of features, *N*_*p*_ represents the number of parallel reservoirs, and *N*_*y*_ represents the number of labels. The matrix size on the MRC is comparable to that on the standard RC, involving three matrix multiplication processes in the reservoir state layer, reservoir state representation, and readout layer, all of which employ linear regression. Following the reservoir state layer, a timestep reduction from 203–198 occurs because the δ value is set to five.

The ESN and grouped ESN differ from MRC primarily in one hyperparameter. ESN shares the same leak rate and a single reservoir, mirroring groupedESN. To align the reservoir nodes with the MRC and grouped ESN, we establish reservoir sizes of 300 and 510 for the ESN. Conversely, grouped ESN maintains the same leak rate but features two and three reservoirs, akin to MRC. We determine the optimal leakage rate for groupedESN to be 0.9.

**Fig 8:**
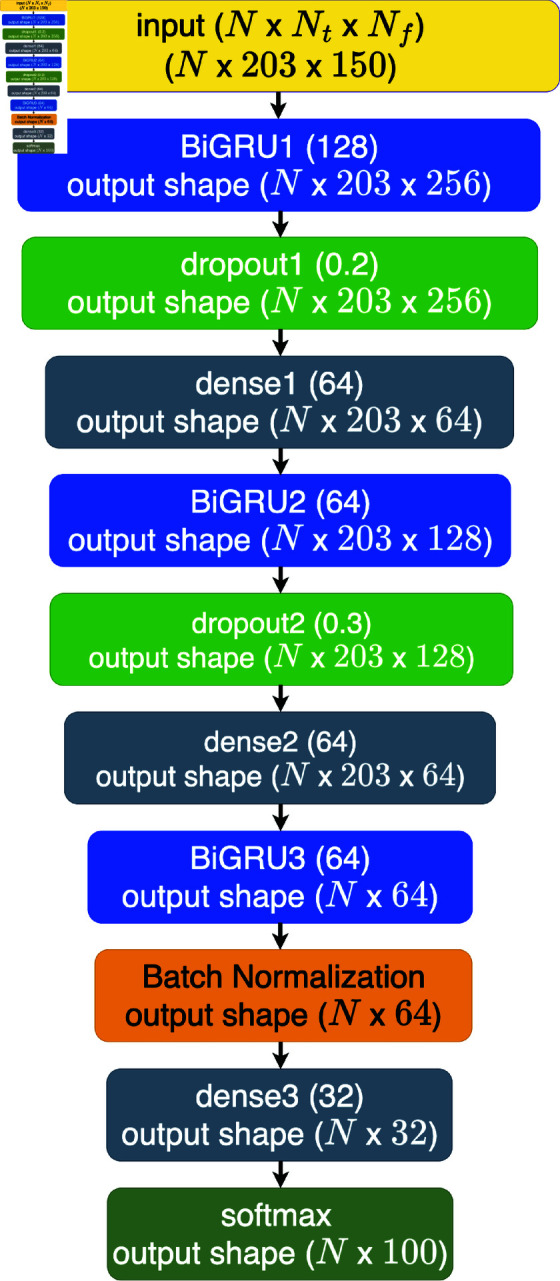
Architecture of BiGRU.

**Fig 9:**
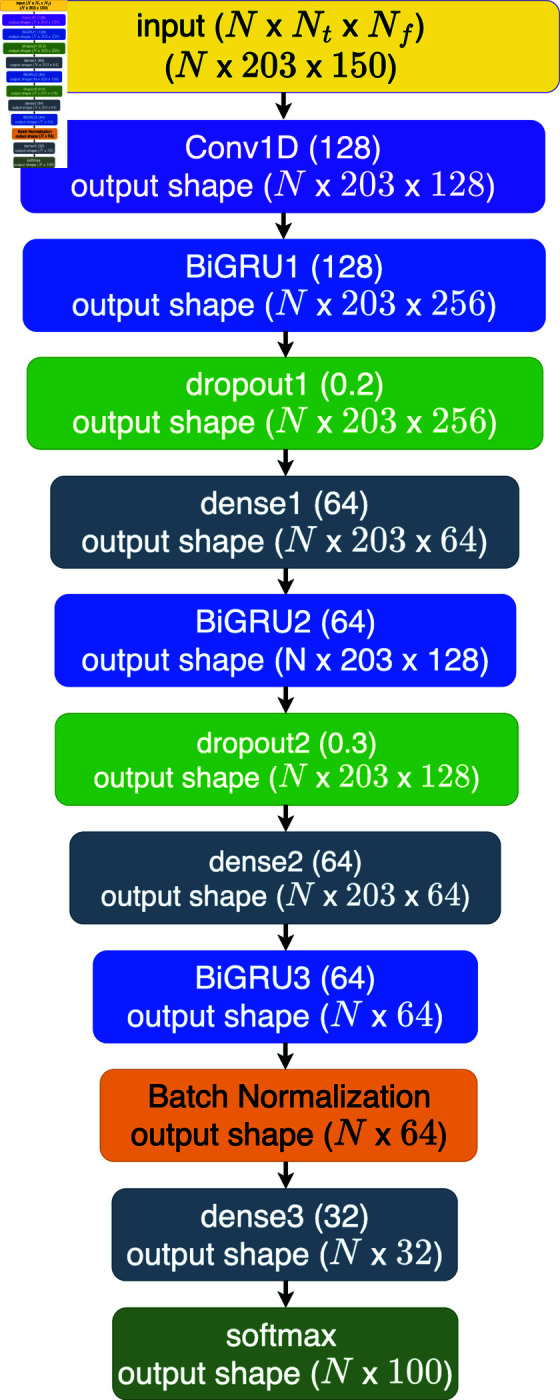
Architecture of Conv1D+BiGRU.

The proposed method underwent a comparative analysis with two deep learning approaches: the bidirectional gated recurrent unit (BiGRU) and one-dimensional convolution (Conv1D) combined with the BiGRU, denoted as Conv1D+BiGRU. The selection of the BiGRU as a benchmark algorithm is grounded in compelling findings from Subramanian’s research [12]. The BiGRU architecture encompasses nine layers, featuring three GRU layers, one batch normalization layer, two dropouts with ratios of 0.2 and 0.3, and three dense layers. The training was conducted over 150 epochs with a learning rate of 10^−4^, utilizing Adam optimization with exponential decay rates of 0.9 and 0.999. The BiGRU architecture is visually depicted in Fig 8. Fig 9 illustrates the Conv1D+BiGRU layer, which is absent in the BiGRU architecture. The inclusion of Conv1D is motivated by the temporal nature of the data, which are organized as time series with each row corresponding to a timestep. The output shapes for each layer in the architectures are displayed in both figures. The dimensions *N*, *N*_*t*_, and *N*_*f*_ represent the number of samples, timesteps, and features, respectively. Notably, the BiGRU3 (64) layer outputs a two-dimensional shape because the network returns the final cell state without the input sequence. This final state is comprehensive in features, facilitating label prediction from the input data.

**Fig 10:**
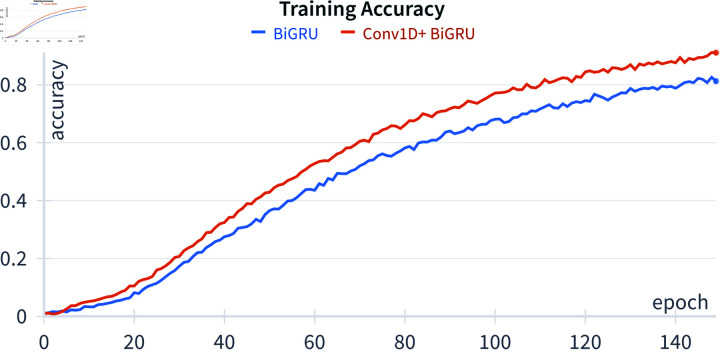
Training accuracy.

**Fig 11:**
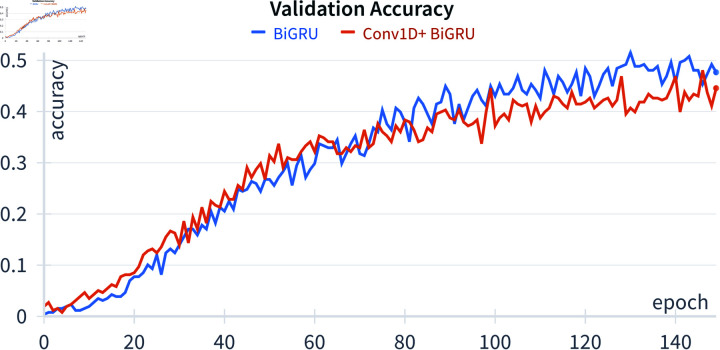
Validation accuracy.

In accordance with the aforementioned experimental setup, the achieved accuracy over 150 epochs is depicted in Figs 10 and 11. Both the BiGRU and Conv1D+BiGRU exhibit a continual improvement in accuracy on both training and validation data throughout the epochs, indicating effective learning from the dataset. Notably, an in-depth analysis reveals that, even before completing the 150 epochs, both algorithms demonstrate superior performance. In light of this observation, the model’s optimal accuracy is selected as the criterion for predicting test data in this study. Moreover, the reservoir algorithm’s processing is notably more straightforward than that of deep learning algorithms. In this algorithm, only the final layer, referred to as the readout layer, undergoes weight updates via Eq 18. Importantly, the training of the reservoir algorithm is a one-time process.

The experimental scenarios are divided into three parts. First, a sensitivity analysis of the leak rate optimized with Optuna was performed. A comparison of the SLR performance of the deep RNN and ESN-based algorithms was then carried out on three types of extracted features. The first type of feature was extracted without normalization. The second type of feature was normalized based on the shoulder as a reference point. The third type of feature was normalized based on the nose as a reference point. In the third scenario, the optimal results from the second scenario were selected and then compared with those of the existing SLR algorithm.

### Experimental results

**Fig 12:**
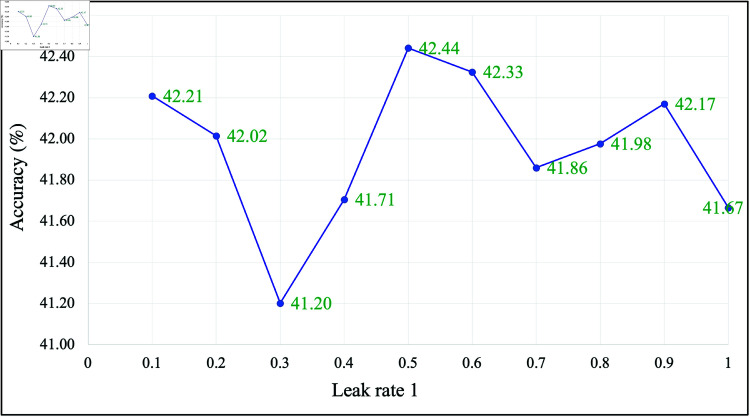
Impact of the leak rate on the SLR accuracy.

The sensitivity analysis conducted in this study aimed to validate the leak rate values suggested by Optuna. In this scenario, the feature used was an extracted feature without normalization. The results are presented in Fig 12, where the accuracy variation across different leak rates can be observed. The figure clearly shows that the accuracy differences across various leak rates were not substantial, indicating that the model remains relatively stable within the tested range. Optuna-suggested leak rates of 0.9, 0.8, and 0.6, which achieved accuracies of 42.17%, 41.98%, and 42.33%, respectively. The highest recorded accuracy was 42.44% at a leak rate of 0.5, showing a 0.27% difference from the Optuna-selected 0.9 leak rate.

These results suggest that Optuna’s selection is reasonable and falls within a stable region. However, the highest accuracy did not occur at the exact Optuna-suggested values, indicating that slight adjustments in the leak rate may further enhance performance. Given the minor fluctuations in accuracy (all within 1.24% of the peak value), it can be concluded that the model is not highly sensitive to variations in the leak rate within this range.

**Fig 13:**
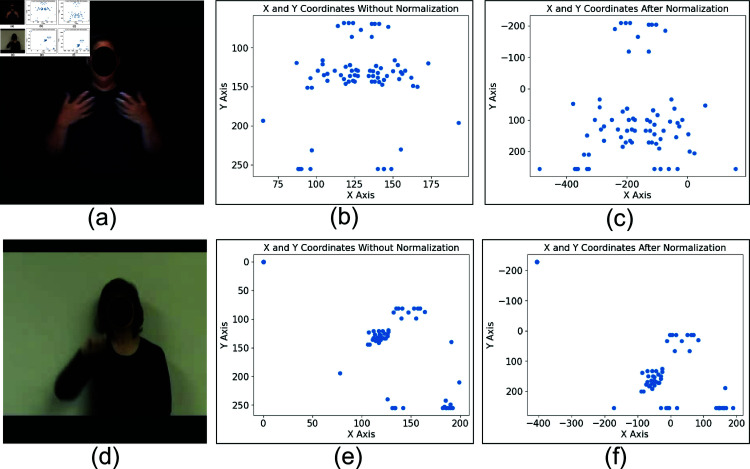
Illustration of (a) A single frame from “accident” sign, (b) A plot of “accident” keypoint without normalization, (c) A plot of “accident” keypoint after normalization, (d) A single frame from “apple” sign, (e) a plot of “apple” keypoint without normalization, and (f) A plot of “apple” keypoint after normalization.

The second experimental scenario was concerned with a comparison of the accuracy of SLR from deep RNN and ESN-based algorithms. A summary of the experimental results is presented in Table 3, which shows the recognition performance without normalization. Additionally, Tables 4 and 5 display the recognition performance via normalization with nose and shoulder as reference points. The normalization is computed via Eqs 22 and (27) for nose normalization and shoulder normalization, respectively. In these tables, Acc refers to accuracy, and SD indicates the standard deviation. The average training and inference times are represented in mm:ss.ms, which means minutes, seconds, and microseconds. The impact of nose normalization is visually depicted in Fig 13. The normalization process involves shifting based on the nose position and scaling of the original keypoints, as illustrated in Figs. 13(b) and 13(e). These images reveal distinct distributions of keypoints due to variations in signer positions and postures. Following normalization, the keypoint distributions become comparable, as evident in Figs. 13(c) and 13(f).

**Table 3:** Comparison of recognition performance without normalization.

Method	Acc±SD (%)	Average Training Time (mm:ss.ms)	Average Inference Time (mm:ss.ms)
BiGRU	35.74 ± 3.38	33:59.9	**00:00.1**
Conv1D+BiGRU	29.65 ± 2.72	35:27.6	**00:00.1**
ESN300 reservoir	42.21 ± 1.02	00:58.9	00:06.2
ESN510 reservoir	46.16 ± 0.48	02:11.3	00:09.4
groupedESN [34] 150*2 reservoir	42.25 ± 0.49	**00:54.4**	00:05.5
groupedESN [34] 255*2 reservoir	45.66 ± 0.40	01:56.5	00:09.2
groupedESN [34] 100*3 reservoir	42.48 ± 1.42	00:57.9	00:05.6
groupedESN [34] 170*3 reservoir	46.71 ± 0.53	01:53.9	00:09.3
MRC 150*2 reservoir	42.56 ± 1.43	00:54.8	00:05.5
MRC 255*2 reservoir	46.55 ± 0.63	02:04.6	00:09.6
MRC 100*3 reservoir	44.81 ± 0.87	00:54.7	00:05.4
MRC 170*3 reservoir	**47.64** ± **0.75**	02:00.2	00:10.7

**Table 4:** Comparison of SLR performance using normalization with both shoulders as reference points.

Method	Acc±SD(%)	Average Training Time (mm:ss.ms)	Average Inference Time (mm:ss.ms)
BiGRU	46.94 ± 2.15	36:44.5	**00:00.1**
Conv1D + BiGRU	40.54 ± 1.14	39:17.9	00:00.2
ESN 300 reservoir	56.67 ± 0.50	01:17.8	00:06.4
ESN 510 reservoir	56.01 ± 1.27	02:05.0	00:09.3
groupedESN [34] 150*2 reservoir	**56.82** ± **1.32**	01:12.7	00:06.4
groupedESN [34] 255*2 reservoir	54.96 ± 1.19	02:00.6	00:09.3
groupedESN [34] 100*3 reservoir	56.09 ± 1.71	01:14.0	00:06.3
groupedESN [34] 170*3 reservoir	55.50 ± 0.58	02:01.4	00:09.8
MRC 150*2 reservoir	55.97 ± 0.94	**01:12.0**	00:05.9
MRC 255*2 reservoir	55.31 ± 1.29	02:03.0	00:09.0
MRC 100*3 reservoir	56.43 ± 0.83	01:15.7	00:05.9
MRC 170*3 reservoir	55.58 ± 0.33	01:58.2	00:09.1

**Table 5:** Comparison of SLR performance using normalization with a nose as a reference point.

Method	Acc±SD(%)	Average Training Time (mm:ss.ms)	Average Inference Time (mm:ss.ms)
BiGRU	50.36 ± 1.41	33:54.1	**00:00.1**
Conv1D + BiGRU	46.59 ± 2.53	35:28.1	**00:00.1**
ESN 300 reservoir	58.64 ± 1.56	00:58.8	00:05.1
ESN 510 reservoir	58.29 ± 1.21	02:23.1	00:09.9
groupedESN [34] 150*2 reservoir	58.45 ± 1.31	00:52.6	00:05.5
groupedESN [34] 255*2 reservoir	58.26 ± 0.84	02:06.1	00:09.1
groupedESN [34] 100*3 reservoir	58.68 ± 1.13	00:53.5	00:05.2
groupedESN [34] 170*3 reservoir	58.45 ± 0.73	01:59.5	00:09.4
MRC 150*2 reservoir	59.42 ± 1.09	00:53.4	00:04.9
MRC 255*2 reservoir	59.42 ± 1.27	02:04.1	00:09.0
**MRC 100** * **3 reservoir**	**60.35** ± **1.52**	**00:52.7**	00:05.2
MRC 170*3 reservoir	58.37 ± 1.18	02:01.8	00:09.4

The experimental results revealed that normalizing significantly improved the recognition accuracy across all the models. From Tables 4 and 5, nose-based normalization outperforms shoulder-based normalization. For example, MRC100*3 achieved 44.81% accuracy without normalization, 56.43% accuracy with shoulder normalization, and 60.35% accuracy with nose normalization, reflecting an improvement of approximately 15.54 points. Similarly, BiGRU’s accuracy increases from 35.74% without normalization to 46.94% with shoulder normalization and 50.36% with nose normalization, whereas Conv1D+BiGRU improves from 29.65% to 40.54% with shoulder normalization and 46.59% with nose normalization. This suggests that normalization enhances the spatial representation, enabling models to better capture the dynamic patterns of sign language gestures. Guided by these findings, normalization was employed in subsequent experiments to optimize model performance.

Five iterations were used in the experiments, with the aim of scrutinizing the standard deviation (SD) of each algorithm. The SD serves as a metric to gauge the variability in accuracy values obtained during the experiments, with lower values being preferable. For the deep learning algorithm, 150 epochs were employed. The accuracy in each table depicts the average accuracy attained by the algorithm across five training and testing sessions with the best-performing model from each session. Notably, in the case of RC, the last weight is utilized, as updates occur at the final layer via Eq 18.

Among the various configurations tested, the MRC exhibited the highest accuracy with 300 reservoir nodes, which is three parallel reservoirs with 100 nodes, achieving a notable 60.35%, coupled with a commendably low SD of 1.52%, as detailed in Table 5. Notably, MRC exhibited superior accuracy compared with its deep learning counterparts, particularly the BiGRU and Conv1D+BiGRU. Upon scrutinizing MRC’s accuracy against ESN and groupedESN, MRC consistently demonstrated superior performance, as exemplified by MRC300 and MRC510. For example, the MRC100*3 configuration achieved an accuracy that was 1.71 points higher than that of ESN300, 1.9 points higher than that of groupedESN150*2 and 1.67 points higher than that of groupedESN150*3. However, notably, in one instance, the MRC170*3 configuration did not outperform the groupedESN170*3 configuration, although it did exceed both the groupedESN255*2 and ESN510 configurations. Overall, the arrangement of 300 reservoir nodes beats 510 nodes via an identical approach. This emphasizes the importance of selecting the number of reservoir nodes for an ESN-based model. Larger reservoir sizes do not necessarily guarantee superior performance in ESN-based models. Having too many nodes can negatively impact the ability of the model to effectively distinguish between features.

Significant discrepancies in training times in Table 5 were observed between the ESN, MRC, and groupedESN approaches compared with the deep learning method. The BiGRU and Conv1D+BiGRU models took 33:54.1 and 35:28.1 minutes, respectively, whereas the fastest ESN-based model, such as MRC100*3, completed training in 0:52.7 seconds. This demonstrates the advantage of the ESN-based method in terms of computational efficiency during training. Notably, the ESN, MRC, and groupedESN exhibited comparable training times when equivalent reservoir sizes were employed. For example, ESN510 finished training at 2:23.1 minutes, whereas GroupedESN255*2 required 2:06.1 minutes, and MRC170*3 achieved 2:01.8 minutes, indicating that the parallel reservoir did not increase the training time.

Furthermore, all algorithms, including deep learning, achieved remarkably fast processing times, thereby demonstrating their potential for real-time applications in SLR. Both the BiGRU and Conv1D+BiGRU had negligible inference times of 00:00.1 s, but the ESN-based models such as MRC100*3 had slightly greater inference times but still had efficient durations of 00:05.2 s. The inference times across the ESN, grouped ESN, and MRC were all less than 10 s.

Overall, MRC100*3 demonstrated the best balance between performance and computational efficiency, attaining the highest accuracy with a minimal training period and rapid inference time. These findings render MRC ideal for tasks that necessitate rapid model updates and real-time recognition.

In the final scenario, a comparative analysis was performed between our proposed method and existing algorithms, and all of these approaches use deep learning. Table 6 presents a comprehensive overview of the recognition performance, where accuracy (Acc) serves as the metric for evaluating correctness in dataset recognition, considering top-k accuracy, including top-1, top-5, and top-10. The average training time is reported in hours, minutes, seconds, and microseconds (hh:mm:ss.ms), whereas the inference time is recorded in minutes, seconds, and microseconds (mm:ss.ms). Additionally, the “Device" column indicates whether the program was executed on a GPU or a CPU. The analysis was conducted via the available code from Li *et al*. [9] for our analysis.

**Table 6:** Accuracy comparison of different approaches on WLASL100.

Method	Acc top-1(%)	Acc top-5(%)	Acc top-10(%)	Training Time hh:mm:ss.ms	Inference Time mm:ss.ms	Device
Pose-TGCN [9]	55.43	78.68	87.60	00:38:18.9	**00:04.2**	GPU
Pose-GRU [9]	46.51	76.74	85.66	-	-	-
I3D [9]	**65.89**	84.11	89.92	20:13:42.5	00:12.5	GPU
MOPGRU [11]	63.18	-	-	-	-	-
**MRC**	60.35	**84.65**	**91.51**	**00:00:52.7**	00:05.2	**CPU**

The results demonstrate that MRC achieves competitive performance while significantly reducing training time. Despite I3D attaining the highest top-1 accuracy, it comes at the cost of prolonged training and inference times, making it computationally expensive. By contrast, MRC achieves the best top-5 and top-10 accuracies when training in less than one minute, highlighting its efficiency. Additionally, MRC is the only approach that operates entirely on a CPU, making it more accessible than GPU-dependent models. Pose-TGCN achieves solid performance but is slightly outperformed by MRC in terms of the top-5 and top-10 accuracies. Pose-GRU exhibits lower accuracy than the other methods, whereas MOPGRU shows promising performance but lacks complete benchmarking data. These findings suggest that MRC provides a highly efficient and practical alternative for sign language recognition on the WLASL100 dataset.

The algorithms under scrutiny include Pose-TGCN, Pose-GRU, I3D, MOPGRU, and MRC. I3D achieved the highest top-1 accuracy, with a score of 65.89%, followed by the MOPGRU, which achieved a score of 63.18%. Our proposed MRC secured the third-highest accuracy, reaching 60.35%, which surpassed the performance of both the Pose-GRU (55.43%) and Pose-TGCN (46.51%). Furthermore, MRC achieved the best top-5 (84.65%) and top-10 (91.51%) accuracies, demonstrating its robustness in recognizing sign language variations. In particular, MRC achieved this competitive performance, with a substantially shorter training time of 00:00:52.7 minutes and an inference time of 00:05.2 seconds while running on a CPU. This underscores the computational efficiency of MRC in comparison with other GPU-dependent models, such as I3D, which requires more than 20 hours of training. This highlights the competitive performance of MRC over deep learning approaches, which are all achieved at an efficient computational cost. Another key advantage of the MRC model is its ability to run on a CPU, as opposed to other models, which require GPU acceleration. This enables MRC to be implemented in low-power and edge computing contexts while maintaining real-time performance.

## Discussion

In the subsection presenting the experimental results, we presented a series of experiments, including sensitivity analysis, normalization, and comparisons with state-of-the-art algorithms. A sensitivity analysis was performed to validate the hyperparameter suggestions from Optuna, and the results confirmed their correctness. Given the inherent variability in the signer’s position and posture across videos, we underscore the importance of normalization in SLR for enhancing accuracy. The primary objective of normalization is to mitigate discrepancies in keypoint positions, ensuring that they exist on comparable scales, thereby diminishing the impact of signer-specific variations in positions and postures. These variations, devoid of distinctiveness, can potentially affect the accuracy of SLR algorithms. In this study, normalization was centered around the nose as a reference point, given its relative stability. Additionally, for comparison, we also applied normalization using the shoulders as a reference point. However, the results showed that normalization based on the nose outperformed the shoulder-based approach. This may be due to the inherent instability of the shoulder position compared with that of the nose because the nose is not affected by hand movement, and the head of the signer is relatively stable. The experimental outcome revealed performance enhancements in all algorithms following keypoint normalization.

We posited that augmenting features and utilizing leak rates could enhance the efficacy of the ESN algorithm, a conjecture supported by the superior performance exhibited by MRC over ESN, groupedESN, and various deep learning algorithms. Notably, the reservoir size in the ESN-based algorithm remained constant across the experiments. The principal distinction arose from the incorporation of distinct leakage rates for each reservoir within the multireservoir structure of the MRC. This leak rate governs the extent to which the prior state is retained, influencing the network’s capacity to store information, as outlined in Eq 28. A higher leak rate implies a diminished impact from historical states, allowing the model to prioritize new inputs.

Our experimental results demonstrated that MRC consistently outperformed ESN-based models, especially when the reservoir size was set to 300 nodes. In one instance, ESN-based approaches with 510 reservoir nodes exhibited performance inferior to that of 300 reservoir nodes. This discrepancy might stem from the increased difficulty in distinguishing more extracted features and misaligning hyperparameter combinations. The performance of ESN-based algorithms is intricately tied to various hyperparameters, including sparsity, the reservoir spectral radius, input weight scaling, and readout weight regularization. This highlights the importance of carefully tuning hyperparameters in ESN-based approaches to avoid reducing the model’s ability to generalize.

All the MRCs and the two ESN-based algorithms exhibit faster training times than their deep learning counterparts. This efficiency stems from the inherently simpler learning process embedded in ESN-based algorithms, as opposed to the deep learning algorithm’s utilization of backpropagation. In the ESN-based paradigm, the learning unfolds solely during the readout phase, employing Eq 18. Compared with their deep learning counterparts, the linear model underpinning the output layer contributes to the lower computational demands of ESN-based algorithms. The expeditious training time assumes significance in SLR for its potential scalability, enabling the training of more extensive datasets within a reasonable timeframe. Moreover, the accelerated training process allows for the implementation of real-time applications by expediting the deployment and enhancement of models.

The inference time of the algorithm remains consistently less than 10 seconds. In general, the inference time of an ESN-based algorithm with an identical reservoir size should exhibit uniformity. However, in this research, slight disparities are observed, likely attributable to variations in computational resources, such as available memory during program execution. Notably, the inference time of the deep learning algorithm surpasses that of the ESN-based model. This phenomenon is potentially attributed to the more efficient implementation of the deep learning framework in comparison to the developed ESN. Upon scrutinizing the processing matrices of each layer in ESN-based algorithms and deep learning, as depicted in Figs 6(b), 8, and 9, a discernible difference emerges. Fig 6(b) illustrates the output matrix shape of the ESN-based algorithm in this study, specifically MRC510, which is equivalent to ESN510 and groupedESN510. This figure provides insight into the ESN-based algorithm’s streamlined processes for predicting labels compared with the more intricate nature of deep learning. For instance, Fig 6(b) shows the complexity of the deep learning approach, which comprises three layers of bidirectional gated recurrent units (BiGRU: BiGRU1, BiGRU2, and BiGRU3) housing numerous BiGRU cells. Each BiGRU cell, in turn, encompasses four independently functioning gates, operating both forward and backward. The finding that ESN processes fewer matrices than deep learning underscores the former’s efficiency in demanding fewer computational resources than its deep learning counterpart does.

A comparison was conducted between the proposed method and other approaches, including Pose-TGCN, Pose-GRU, I3D, and MOPGRU. All of the comparison algorithms employed a deep learning architecture to develop the SLR system and utilized 2D keypoints extracted by OpenPose [38] for TGCN, Pose-GRU and I3D, whereas MOPGRU employed MediaPipe, which is similar to the proposed method. In addition, I3D combines spatial and temporal features. The proposed method demonstrated comparable performance to the deep learning approach, which achieved 60.35%, outperforming Pose-TGCN and Pose-GRU. I3D achieved the highest top-1 accuracy at 65.89% because I3D contains high model capacity owing to its high number of parameters. Therefore, I3D relies on extensive GPU training, and the training time exceeds 20 h on a GPU, which limits its practical applicability in the context of device training. By contrast, MRC achieved the best accuracy in the top-5 (84. 65%) and the top 10 (91. 51%), demonstrating its ability to capture the features of sign language. Our algorithm leveraged the dynamics in the reservoir layer to represent the input feature, and the multiple reservoir model enabled the extraction of features with greater variation than a standard reservoir. The proposed method, MRC, has been shown to achieve a balance between efficiency and accuracy. MRC demonstrated that it achieved a top-1 accuracy of 60.35% with a training time of 52.7 s on a CPU, thus substantiating its feasibility for edge computing.

## Conclusions

In this study, we explored the performance of ESNs through the standard ESN, MRC, and groupedESN approaches. The findings of this study indicate that the proposed MRC method, which incorporates various leak rates, enhances feature representation, enabling the network to acquire a more profound understanding than the standard ESN. Consequently, it demonstrates competitive performance when juxtaposed with deep learning approaches, achieving 60.35% top-1 accuracy, 84.65% top-5 accuracy, and 91.51% top-10 accuracy. Moreover, MRC has efficiency advantages, requiring less training time and fewer resources than deep learning does, which is attributed to its streamlined processes and reduced number of matrix computations within the ESN. This implies the feasibility of deploying RCs on portable devices with constrained computational resources, such as limited RAM and processors.

Although the results are promising, they fall short of achieving state-of-the-art benchmarks. Future research efforts will focus on refining accuracy by employing a modified ESN in conjunction with other machine learning methods. Additionally, we aim to implement multiple reservoirs on embedded hardware, such as field-programmable gate arrays (FPGA) [39–41], and explore physical RC. This approach will empower users to carry the system portably and deploy it as needed.
